# Observation of the thermal influenced quantum behaviour of water near a solid interface

**DOI:** 10.1038/s41598-018-24886-y

**Published:** 2018-05-03

**Authors:** Hongkee Yoon, Byoung Jip Yoon

**Affiliations:** 1 Department of Physics, Korea Advanced Institute of Science and Technology, 291 Daehak-ro, Yuseong-gu, Daejeon, 34141 Korea; 2 Department of Chemistry, Gangneung-Wonju National University, 7 Jukheon-gil, Gangneung-si, Gangwon-do 25457 Korea

## Abstract

Water is essential for life. However, the structure and properties of water are still not well understood. It has been introduced that anomalies are in vicinal water near solid interfaces. We performed capillary flow experiments on water with a silica colloid sample using a high-performance liquid chromatography (HPLC) system by accurately varying the temperature and analysed the peak shape rigorously. We obtained a novel anomalous temperature spectrum from the peak-shape analysis. Here we report the observed distinct specific anomalous temperature (SAT) behaviour in vicinal water at silica interface. The anomaly appeared in the viscous force that was derived from a relationship between the shape of the HPLC peak and the velocity profile for the capillary flow. The observations were highly reproducible, and we conclude that the SAT is related to the quantum mechanical behaviour of water, in agreement of the characteristic acceptance of thermal displacement according to the Franck-Condon principle. We performed the same experiments using heavy water and water mixed with a small amount of methanol, and the results support the quantum phenomenological origin.

## Introduction

Water is common on earth and essential for life. However, the structure and properties of liquid water are still not well understood, and water remains a mysterious subject^1–3^ and continued issues^4^. To describe the structure of liquid water, quantum mechanical studies have been performed in various aspects, usually at a single temperature. The quantum mechanical treatments improved the classical results^5–7^, and quantum phenomena were obvious in confined water^8–10^. Recent issues are also to investigate the nuclear quantum effects^11–13^. However, the improvements have a certain distance from solving the mysteries of water for life. It has been introduced that water confined between quartz plates also exhibits anomalous behaviours at the approximate multiple of 15 °C^14,15^, and Drost-Hansen extended this idea to the room that the vicinal water anomaly (VWA) near solid surfaces has an important role in cell-functions and life^16–18^. However, no clear experimental evidences have been provided^18–21^, and the anomalous behaviour has been questioned^22^. The VWA has been explained as a quantum mechanical phenomenon by modelling the relationship between the thermal wavelength and the small molecular free volume space of water^23^. Obtaining apparent VWA has been difficult in previous experiments by measuring the heat capacity of water in silica pores^20,21^, probably because VWA is a quantum phenomenon that appears at a very short range of solid-water interface and is hard to be observed in a macroscopic variable such as the heat capacity.

In this study, we observed the anomalous temperature spectrum, probably related to VWA, by examining the laminar flow of water with a silica colloid sample flowing through a capillary using a high-performance liquid chromatography (HPLC) system (Supplementary I) and accurately varying the temperature. This new application of the HPLC system is different from the general purpose of chromatography of detecting only the peak positions. The shape of an HPLC peak reflects the flow of an injected sample with differently developed speeds layer-by-layer through the capillary. The characteristics of the flow was analysed from the peak shape (see Methods). The sample contains a minute amount of thiourea so that it can be monitored by a UV detector and exhibits its own distinguished peak shape depending on the temperature and the pump speed. We used a silica colloid sample for examining the interfacial water. The silica colloids are easily dispersed in water and hydrogen bonding sites are on the surface of silica. The structure of interfacial water near silica colloid represents a wide distribution of strong hydrogen bonds^24^.

**Figure 1:**
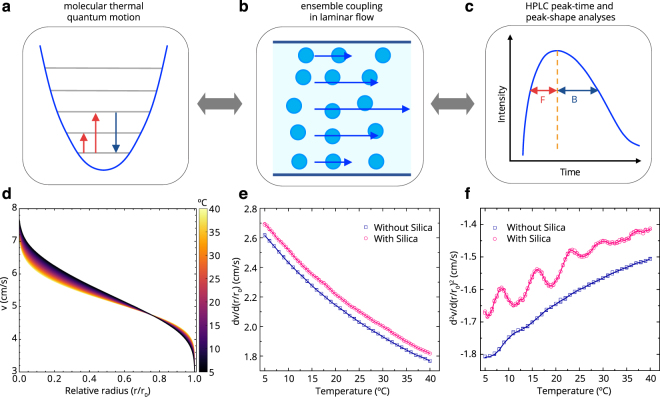
Observation of the quantum behaviour and velocity profiles. (**a**–**c**) The conception of the observation of the molecular quantum behaviour in a macroscopic scale of an HPLC peak through the coupling in laminar flow. An asymmetric HPLC peak shape is represented in (c). The dashed vertical line in (c) is at the peak maximum. (**d**) The velocity profiles (without colloidal silica) calculated along the capillary radius (*r*_0_ = 0.25 mm) at various temperatures are shown as coloured lines. (**e**) The first derivative (*dv*/*dr*) is at *r*/*r*_0_ = 0.67. The negative sign was applied, since the velocity decreases from the capillary centreline to the wall. (**f**) The second derivative (*d*^2^*v*/*dr*^2^) is at *r*/*r*_0_ = 0.67. Anomalies upon temperature appear in the up-peaks. The overlapping three data points at the same temperature in (e,f) signifies the high reproducibility of the experiment and analysis.

Quantum phenomena are observed through coupling of hybrid systems^25,26^ and at room temperature^27^. In Fig. 1a–c, we represented the conception of the observation of quantum phenomena in a macroscopic scale by analysing the HPLC peak that implicates the ensemble coupling of molecular quantum behaviour.

## Results and Discussion

### Anomalous temperatures from velocity profile of the capillary flow

We derived a relationship between the shape of the HPLC peak and the velocity as a function of the capillary radius (see Methods). In Fig. 1d, the calculated velocities with respect to the capillary radius were shown at various temperatures for the pure H_2_O sample. The calculated velocity profiles were different from a parabolic form that is commonly considered in a pipe flow for which the velocity derivative at the centre is zero (Supplementary II). The slope of the flow velocity along the capillary radius was generally flattened with increasing temperature or with decreasing viscosity, and the overall pump speed was the same for all temperatures. Therefore, the coloured lines were twisted in Fig. 1d.

The Reynolds number (Re) of the system for which we performed the experiments is approximately 20, which is sufficiently smaller than the value for the turbulent flow (Re > 2300). Hence, a substantial turbulence was not expected in our experiments. From the calculated velocity profile, we obtained the velocity derivatives with respect to the radius. The velocity derivatives at the same radius around the twisted position in Fig. 1d, and where the HPLC peak was near the maximum (Supplementary Fig. S2a), were plotted against the accurately controlled temperature for the samples with and without silica colloid in Fig. 1e,f. The temperatures were changed by 1.0 °C for the sample without silica colloid and by 0.5 °C for the sample with silica.

The first derivative (*dv*/*dr*; Fig. 1e) which is related to the shear force^28^ did not show any anomalous behaviour. However, interestingly, the anomalous behaviour of consecutive peaks upon the temperature appeared in the second derivative (*d*^2^*v*/*dr*^2^; Fig. 1f) for the sample with silica colloid, which is related to the viscous force^28^. Anomalous temperatures (ATs) were present in the up-peaks that are far off the line of without silica. The measured velocity profiles with respect to the capillary radius and temperature also enable other various data analyses for the capillary flow. We formulated an equation for describing the velocity profile by separating the flow into a draw force term and a drag force term (Supplementary II); the anomalous behaviour reappeared clearly in the draw force term even in the first derivative (Supplementary Fig. S4). In the velocity analyses, no anomaly occurred without silica colloid in the sample, either.

### Consistency of the anomalous temperatures from peak-shape analysis

**Figure 2:**
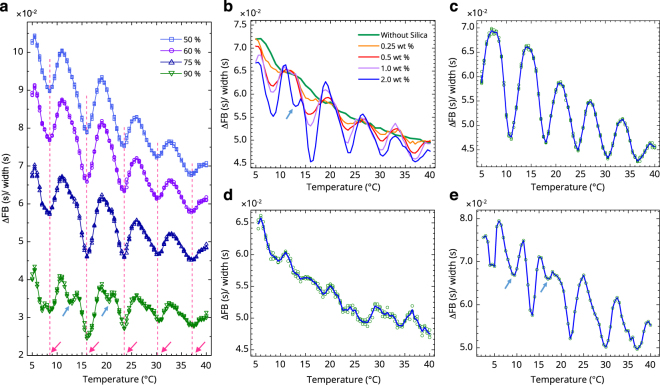
Anomalous temperature spectra. (**a**) The ratio of the difference between front width (F) and back (B) (see Fig. 1c) relative to the full width, i.e., (B − F)/(B + F) at various peak-height positions at 50, 60, 75, and 90% of the HPLC peak at a pump speed of 0.75 mL/min and a silica colloid concentration of 1.5 wt%. The red arrows indicate ATs. (**b**) The same as in (a) but the concentrations of the silica colloid were 0, 0.25, 0.5, 1.0, and 2.0 wt% in the sample. (**c**,**d**) The same as in (a) but at a pump speed of (c) 0.85 mL/min (anomalous temperatures are different from those in (a), and (d) 0.6 mL/min (the peaks were scarcely observed and not in a regular manner). (**e**) The same as in (a) but the carrier and sample were D_2_O that shows an intense behaviour with a silica colloid concentration of 1.2 wt%; the peaks are deep and clear even with a smaller silica colloid concentration. The peak-height position is at 75% in (b–e). The blue arrows in (a,b,e) indicate shoulder peaks to be discussed.

Not only the velocity analysis, but we also examined the peak shapes in detail. The HPLC peaks were all asymmetric^29^ when the shapes of the front and back from the top were compared (see Fig. 1c). The peak widths and asymmetries depend on flow characteristics in liquid chromatography^30^. We examined the difference between the widths of the front (F) and the back (B) at several HPLC peak height positions. In Fig. 2a, the ratio of the difference between the widths of the front and the back relative to the full width of the HPLC peak, i.e., (B − F)/(B + F) was plotted against temperature. This ratio is little affected by the retention time in chromatography^30,31^ or by the constitution of the capillary in this experiment. The results showed a pattern of a temperature spectrum that appeared in the distinct down-peaks at consecutive temperatures. The differences between the widths of the front and the back were considerably small, but the reproducibility was high, as indicated by the small scattering of the data points in the figure. Disregarding the positions of the HPLC peak heights, the down-peaks appeared at the same ATs when the sample contained colloidal silica.

**Figure 3:**
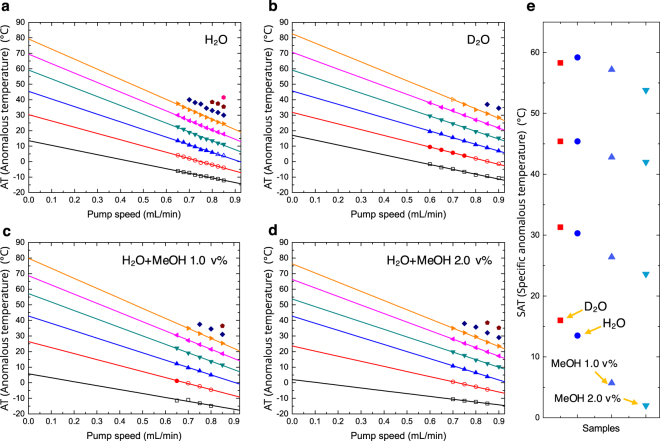
Extrapolated anomalous temperatures. Experiments were performed at various pump speeds, and the results were extrapolated to a zero pump speed for (**a**) H_2_O and for (**b**) D_2_O. (**c**) H_2_O with 1.0 v% MeOH; the SATs (ATs at a zero pump speed) were 6, 26, 43, and 57 °C. (**d**) H_2_O with 2.0 v% MeOH; the SATs were 2, 24, 42, and 54 °C. The open symbols were extrapolated with quadratic functions along the anomalous temperatures obtained in the experiments at the same pump speed, whereas the extrapolations along the pump speed were performed using linear functions. (**e**) The overall plot of the SATs for the samples in (a–d). The factors for describing the slopes are formulated in the text.

In Fig. 2b, the same results are plotted for different concentrations of silica colloid; the ATs did not change. However, the depths of the down-peaks increased with increasing silica concentration. When no silica was present, no peaks appeared and thus no anomalous behaviour was observed as in the velocity analysis. Therefore, we believe that the phenomena are due to the vicinal water near the silica solid surface, and a result of the combined and cumulative effects of the numerous thin layers of the flow through a long capillary as an ensemble coupling illustrated in Fig. 1b. (The effect of capillary-surface layer is not significant.) The ATs showing the aforementioned down-peaks occurred when the pump speed was at 0.75 mL/min. The series of ATs of the down-peaks altered when the pump speed was changed. In Fig. 3a, the ATs at different pump speeds were shown. At a high pump speed, the AT decreased and the intervals between the ATs became narrower. Although the peaks were unclear in the experiments at low pump speeds (below 0.6 mL/min as shown in Fig. 2d) and no information is obtainable at a zero pump speed or without flow, we extrapolated the ATs to a zero pump speed (Supplementary III) and determined that the values were 14, 30, 45, and 59 °C. These specific anomalous temperatures (SATs) not depending on the pump speed are similar to the Drost-Hansen temperatures (15, 30, 45, and 60 °C) at which the viscosity is higher^15,17^.

The down-peak indicates that the interaction between the layers of the flow is stronger (Supplementary II) at the ATs. The viscosity of water with silica colloid is higher than that of pure water. The HPLC peak full widths (Supplementary Fig. S2b) were examined; the widths were wider at the ATs, and this implies that the viscosity is higher at the ATs (Supplementary II).

### Quantum phenomena according to the commensurate thermal displacement

The previous suggestion^23^ for VWA that the anomaly is due to the resonance matching of several multiples of thermal half-wavelength to the small free volume space is interesting. Moreover, if the VWA originates from the quantum mechanical behaviour, heavy water (D_2_O) should exhibit the anomalous behaviour expected^23^ at 19, 32, 44, and 56 °C. We performed the same experiment with D_2_O (an example is in Fig. 2e), and the extrapolated SATs for the stationary state were 16, 31, 45, and 58 °C as shown in Fig. 3e. The expected and calculated free volumes of water were reasonable^32^, and the quantum phenomena were specifically due to the motion in the small free volume space of water; however the observed SATs for D_2_O were slightly off the predicted values with the previous model, and the energy gap of 15 °C in the free volume space is a little narrow for defining quantum energy levels and considering the broad thermal energy distribution. Therefore, the model is less appropriate for describing the observations. (When the previous resonance matching model was adoptable, the thermal energy distribution may differ from the Boltzmann distribution.) Regarding the ATs, we propose a similar quantum mechanical concept that also assumes the thermal displacement of water molecules in small free volume space.

**Figure 4:**
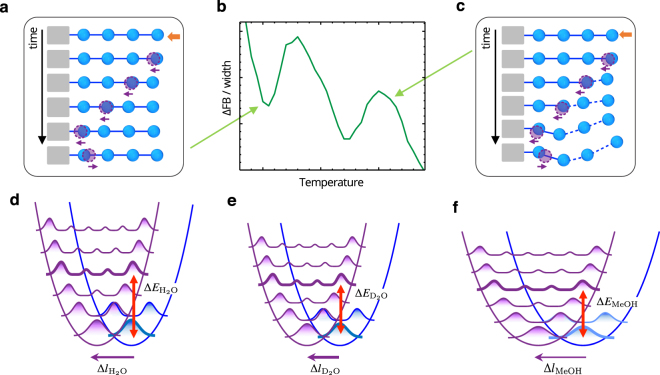
Proposed origin of the observed anomalous behaviour. (**a**) Illustration of the consecutively commensurate displacements accepted by the Franck-Condon principle as in (d) for the 1-D linear water cluster moving into free volume space by an impact near a solid wall exhibiting the down-peaks in (b). (**b**) A capture from Fig. 2. (**c**) As in (a) but the displacement and energy are not accepted by the Franck-Condon principle and dissipated. (**d**,**e**) Accepted energy (Δ*E*) and displacement (Δ*l*) by the Franck-Condon principle for (d) H_2_O and (e) D_2_O. The displacement shown is an example of the overlap between the initial ground level (blue) and the 3rd excited level (purple) of a displaced state and is about 0.2 Å for H_2_O. The energy levels are lower for D_2_O because of the heavier mass and the energy gaps are also smaller. It requires less energy (${\rm{\Delta }}{E}_{{D}_{2}O} < {\rm{\Delta }}{E}_{{H}_{2}O}$). Another overlap occurs between the first excited levels of initial and displaced states. (**f**) The same overlap as in (d) but with an arbitrarily broadened potential of a less-structured network for water-methanol mixture. It requires less energy (${\rm{\Delta }}{E}_{MeOH} < {\rm{\Delta }}{E}_{{H}_{2}O}$) but longer displacement (${\rm{\Delta }}{l}_{MeOH} > {\rm{\Delta }}{l}_{{H}_{2}O}$). The impact energy in (a or c) is the thermal energy only at a zero pump speed, however the energy at high pump speeds is added due to the HPLC pump or the force by the velocity gradient that is sufficient to break the hydrogen bonds. The energy gap is about kT.

Figure 4a illustrates a one-dimensional (1D) linear water model cluster at a solid surface impacted by thermal kinetic energy for which the displacements are commensurate by the Franck-Condon principle as shown in Fig. 4d, exhibiting the down-peaks in Fig. 4b. Figure 4d represents the energy levels with large gaps and wave functions (6-fold) that were calculated by numerically solving the 1D Schrödinger equation for the potential formed by neighbouring water molecules with reasonable parameters^33,34^. The accepted movements are stepwise along the parabolic-like potential curve. This displacement is assumed in a larger scale than the molecular vibration and is large enough to move the molecular position. (At this stage, we avoid exact peak assignments for energy level changes.) This properly accepted condition maintains a localized stable structure when the impact is transferred without disturbing the structure. However, in Fig. 4c, the displacement and energy are not accepted by the Franck-Condon principle, and the structure is destroyed and unstable. In Fig. 4a, the ordering occurs at a short range of solid-water interface, but it affects the motion of the whole colloidal silica which makes the interface effect greatly reflected in the capillary flow. In that case, the roughness of colloidal silica surface would affect little on the overall phenomena, since the roughness is defined in a larger scale than molecules.

### Factors for the slope of anomalous temperature along the pump speed

The ATs also depend on the pump speed. We express the ATs in a linear function of the pump speed for a single AT series, as follows;

1 $${\tau }_{0}=\tau (P)-\alpha P,$$

where $\tau (P)$ is the AT in Kelvin as a function of the pump speed, *P*; ${\tau }_{0}$ is the SAT at a zero pump speed, and *α* is the slope.

The thermal displacement is a factor of the thermal kinetic force, and the force, i.e., the 1D pressure, depends linearly on the temperature^35^. A force is also generated between the flow layers by the velocity gradient between them (Supplementary III). This force contributes to the displacement, in combination with the thermal kinetic force along the flow direction. The displacement, Δ*l*_0_, in Fig. 4 is contributed in two terms;

2 $${\rm{\Delta }}{l}_{0}={\rm{\Delta }}{l}^{thermal}+{\rm{\Delta }}{l}^{external},$$

where Δ*l*_0_ is the displacement at a zero pump speed. Here, ${\tau }_{0}$ and Δ*l*_0_ are intrinsic values not depending on the pump speed. In our experiment, Eq. (2) is simply expressed assuming only the linear term with pump speed;

3 $${\rm{\Delta }}{l}_{0}={\rm{\Delta }}{l}^{thermal}+\delta {\rm{\Delta }}{l}^{pump}\,P.$$

Reducing Eqs. (1) and (3) with respect to ${\tau }_{0}$ and Δ*l*_0_, respectively, yields,

4 $$\frac{\tau }{{\tau }_{0}}-\frac{\alpha }{{\tau }_{0}}P=\,\frac{{\rm{\Delta }}{l}^{thermal}}{{\rm{\Delta }}{l}_{0}}\,+\frac{\delta {\rm{\Delta }}{l}^{pump}}{{\rm{\Delta }}{l}_{0}}P,$$

in which the terms are dimensionless. We assume that the thermal displacement is proportional to the temperature^35^. Thus, in Eq. (4), the first terms in the left and right are almost the same and they are cancelled, then we have a relation;

5 $${\rm{\alpha }}\,=-\,{\tau }_{0}\frac{\delta {\rm{\Delta }}{l}^{pump}}{{\rm{\Delta }}{l}_{0}}.$$

Thus the slopes in Fig. 3a–d are negative and are contributed in three factors for simplicity. The slope, *α*, includes the quantum mechanical properties such as ${\tau }_{0}$ and Δ*l*_0_. The factor, $\delta {\rm{\Delta }}{l}^{pump}$, stands for the displacement elongation by the pump speed in the dimension of length/(pump speed) and is related to the molecular interactions.

### Anomalous temperature behaviour according to molecular quantum interactions

#### Isotope quantum effect for heavy water

The patterns of negative slope and larger declining tendency for higher ATs in Fig. 3 reflect equation (5), in general. Most of the differences in properties between H_2_O and D_2_O are explained by quantum effects^12,36–38^. D_2_O has the stronger intermolecular interaction due to the lower zero point energy than H_2_O; therefore, the SATs at a zero pump speed are higher than those of H_2_O at low temperatures (Fig. 3e). And the displacement is less affected by the pump speed for D_2_O than for H_2_O, i.e., $\delta {\rm{\Delta }}{l}_{{D}_{2}O}^{pump}$ is smaller than $\delta {\rm{\Delta }}{l}_{{H}_{2}O}^{pump}$ in Eq. (5). When a heavier mass is applied, lowered energy levels and smaller energy gaps are expected as in Fig. 4e. In this case, a lower energy is required for the wave functions to overlap. Because of the smaller energy gaps, the SATs at high temperatures are low effectively, compared to other higher temperature properties of D_2_O such as the melting point of increase of 4 °C. Since $\delta {\rm{\Delta }}{l}_{{D}_{2}O}^{pump}$ is smaller and ${\tau }_{0}^{{D}_{2}O}$ is effectively lower than those for H_2_O, the declining slope, ${\alpha }_{{D}_{2}O}$ (Fig. 3b) is lower than the slope, ${\alpha }_{{H}_{2}O}$ (Fig. 3a) with increasing SATs (Table 1). The narrow width and high amplitude of the wave functions for the lowered energy levels also result in fine overlaps between the wave functions; thus, D_2_O exhibits more intense spectra of anomalous behaviour in Fig. 2e. These also support that the observations are involved from quantum phenomena^39,40^.

**Table 1:** Slopes, *α* (K/(mL/min)), of the fitted line (AT as a function of pump speed) in Fig. 3 and in the parentheses are the estimated ratios, *γ* (/(mL/min)), of the external displacement to the displacement at a stationary state in Eqs. (2) and (3).

Line number^a^	1	2	3	4	5
H_2_O	−30.1 (0.105)^b^	−40.5 (0.133)	−49.3 (0.155)	−57.1 (0.173)	−61.1 (0.178)
D_2_O	−29.9 (0.103)	−36.6 (0.120)	−42.8 (0.134)	−48.2 (0.145)	−53.8 (0.157)
H_2_O+MeOH 1.0 v%	−25.3 (0.091)	−38.4 (0.128)	−47.2 (0.149)	−54.2 (0.164)	−59.0 (0.173)
H_2_O+MeOH 2.0 v%	−18.0 (0.065)	−32.8 (0.111)	−44.6 (0.141)	−48.6 (0.149)	−54.1 (0.160)

^a^The line numbers from the bottom in each Fig. 3a–d.

^b^For an example, the ratio of this result at a pump speed of 0.7 mL/min is 0.105 × 0.7 = 0.0735.

#### Additional experiments for destructured water network

On the basis of the relationship between the observation and the quantum behaviour, we attempted to destruct water networks by dissolving a small amount of methanol^41^ (Supplementary IV) and then performed the same experiments. Less-structured water has weaker molecular interactions and requires a smaller kinetic force (lower temperature) for a given displacement. This gives the lower SATs, i.e., the smaller ${\tau }_{0}^{MeOH}$ (here, MeOH stands for the water-methanol mixture). Additionally, the shape of the potential is broader in the less-structured network, as shown in Fig. 4f. Thus a longer displacement, i.e., a larger ${\rm{\Delta }}{l}_{0}^{MeOH}$ is required to overlap the wave functions, and more kinetic force is required at the same pump speed. Since the declining slope, *α*, is proportional to smaller ${\tau }_{0}^{MeOH}$ and inversely proportional to larger ${\rm{\Delta }}{l}_{0}^{MeOH}$, the slopes were in the order of ${\alpha }^{{H}_{2}O}$> ${\alpha }^{{H}_{2}O+MeOH1{\rm{v}}{\rm{ \% }}}$> ${\alpha }^{{H}_{2}O+MeOH2{\rm{v}}{\rm{ \% }}}$ in Fig. 3a,c,d. In Table 1, we presented the slopes (*α*) and the ratios (*γ*) of the external displacement (${\rm{\delta }}{\rm{\Delta }}{l}^{pump}P$) contributed to the displacement at a stationary state (${\rm{\Delta }}{l}_{0}$; see Fig. 4). These ratios are estimated using Eq. (5). The ratios were also in the same order of ${\gamma }^{{H}_{2}O}$> ${\gamma }^{{H}_{2}O+MeOH1{\rm{v}}{\rm{ \% }}}$> ${\gamma }^{{H}_{2}O+MeOH2{\rm{v}}{\rm{ \% }}}$ as the slopes, and ${\gamma }^{{D}_{2}O}$ was smaller than ${\gamma }^{{H}_{2}O}$ as discussed above.

### Shoulder peaks in the anomalous temperature spectra

Overlap also appears in Fig. 4d–f, with less probability, between an excited initial level and other levels of the moved position (hot bands in spectroscopy), since the HPLC pump supplies high energies in water networks. Some apparent shoulder peaks were shown in Fig. 2a (90% case, which shows more fine peaks), in Fig. 2b (for high silica concentration), and in Fig. 2e (D_2_O produces fine and intense spectra because of quantum effects as discussed above), which are explained by the less-probable overlaps.

The observed sharp temperature behaviour is explained by quick flipping of the displacement back and forth a number of times and/or by the effect of many-particle statistics. The 6-fold wave functions in Fig. 4d–f show that the amplitudes of wave functions diminish considerably except at the boundary of the potential. These additional observations for heavy isotope and less-structured water-MeOH mixtures are interpreted with the quantum effects from Franck-Condon phenomena and related Eq. (5).

## Conclusions

We observed the temperature dependent anomalous specific behaviour (SAT) of vicinal water at a solid interface by rigorously analysing the peak shapes of the capillary flow obtained using an HPLC system, which is a state of the art technique. The favoured stepwise acceptance of displacement based on the quantum mechanical wave behaviour would be an explanation for the VWA as increasing the dynamical stability of water molecules at a solid surface or a rigid boundary because of ordering of the movement. However, the opposite effects of disordering would be expected in bulk water because the end is not bound but free. This affects the viscosity of bulk water to be lower in the quantum mechanical treatment than the classical^42^. The observed behaviours (ATs) from the experiments are no longer anomalous and no wonder when the origin is elucidated. If the observations are convincing quantum phenomena of water molecules, it is interesting and noticeable that the microscopic quantum behaviours are observed in a macroscopic scale in HPLC peaks. The observation has been possible through the ensemble coupling of laminar flow of quantum nature for which the energy gap is in the magnitude of thermal kinetic energy of kT in the strong hydrogen-bond and one end is fixed, i.e., water at a solid interface, and because the high energy was supplied in water networks by the HPLC pump. Finding the quantum phenomena of water will be a near feature issue, and quantum model helps solve mysteries of water^43^. Even though the difference of the properties of interfacial water upon the temperature is small, the influence in living organisms^44^ might be significant, since the overall multiplied effects are through numerous steps of interface channels in organs. The key to solving the mysteries of water including functions in life^18,23^ may be in a place that we already know but have ignored, possibly in the quantum mechanical wave behaviour that water exhibits in a small free volume space.

## Methods

### Constructing the HPLC system

To examine the detailed flow in the capillary, we developed a system (Supplementary I) consisting of an HPLC pump (Gilson; 305), UV detector (Gilson; 151), and capillary loop (DuPont FEP tubing; 1/16″ OD, 0.50 mm ID, 2 m long). The capillary was rolled onto a round copper block (ca. 8 cm diameter), and its temperature was accurately controlled by Peltier thermoelectric modules located between the copper block and a water chamber. The temperature of the chamber was maintained at the approximate desired value by a circulating thermostat. The temperature of the copper block was monitored with a thermistor, and the DC voltages applied to the Peltier modules were adjusted for heating and cooling by feed-back to the monitored temperature. The temperature was controlled within 0.005 °C or less for a continuous flow. We injected a 10 μL sample, in which a minute amount of thiourea was dissolved, and the peaks were obtained by the UV detector after the samples passed through the capillary. The amount of thiourea (ca. 0.032 wt%) was chosen so that the height of the HPLC peak is around 1 a.u. at a fixed wavelength of 235 nm, where the aqueous thiourea solution gives a maximum UV absorption. The UV absorptions were read at every 0.05 second interval. Colloidal silica (Sigma-Aldrich; 50 wt% suspension, surface area ~140 m^2^/g) of given concentration was mixed in the sample.

### Derivation of the velocity profile from the HPLC peak shape

The shape of an HPLC peak reflects the developed velocity after flowing through a capillary. The relationship between the shape of the HPLC peak and the velocity depending on the radius of the capillary is obtained starting from the following relation,

6 $${\int }_{0}^{\infty }I(t)dt=\pi {r}_{0}^{2}{l}_{0}\rho =\,{I}_{0},$$

where *I*(*t*) is the intensity of the HPLC peak as a function of time, *t*, *r*_0_ is the radius of the capillary, *l*_0_ is the initial length of the injected sample in the capillary, and *ρ* is the density of thiourea. Thus, *I*_0_ is the total amount of the injected sample.

**Figure 5:**
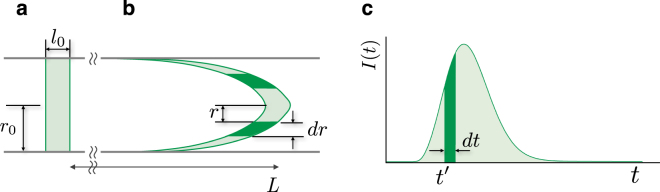
Relationship between the developed sample in the capillary and the HPLC peak. (**a**) The initial injected sample. (**b**) The developed sample after flowing through the capillary. (**c**) The shape of the HPLC peak. The shaded volume in (a,b) corresponds to the shaded area in (c).

At a certain time, *t*′,

7 $$v(r(t^{\prime} ))=L/t^{\prime} ,$$

where *v* is the velocity of the layer that is spaced *r* from the centreline of the capillary and *L* is the length from the injection point to the detector. However, because the route from the end of the capillary to the detector is very narrow, *L* is the length of the capillary itself where the temperature is controlled. We find the following relation (see Fig. 5):

8 $$I(t)dt=2\pi \,r(t)\sigma dr,$$

where $\sigma$ is the surface density of thiourea which is equal to ${l}_{0}\rho$ from Eq. (6). When it is substituted with ${I}_{0}/\pi {r}_{0}^{2}$, Eq. (8) becomes

9 $$\frac{1}{{I}_{0}}I(t)dt=\,\frac{2}{{r}_{0}^{2}}\,r(t)dr.$$

Integrating Eq. (9) gives

10 $$r(t^{\prime} )/{r}_{0}={\{\frac{1}{{I}_{0}}{\int }_{0}^{t^{\prime} }I(t)dt\}}^{1/2}.$$

From Eqs. (7) and (10), $v(r)$ is obtained numerically. The velocity does not depend on the amount or concentration of the injected sample since the right-hand side of Eq. (10) is reduced with *I*_0_. The derivation is based on the assumption that the flow maintains parallel streamlines.

## Electronic supplementary material

Supplementary Information
