# Supplementary material for: Observation of the thermal influenced quantum behaviour of water near a solid interface
Source: Sci Rep. 2018 May 3;8:7016. doi: 10.1038/s41598-018-24886-y (PMC5934364; doi:10.1038/s41598-018-24886-y)
Supplement: Supplementary file 1 — Supplementary Information [file 41598_2018_24886_MOESM1_ESM.pdf]

Supplementary Information

Observation of the thermal influenced quantum behaviour of water near a solid interface

Hongkee Yoon[1] & Byoung Jip Yoon*[2]

[1]Department of Physics, Korea Advanced Institute of Science and Technology, 291 Daehak-ro, Yuseong-gu, Daejeon, 34141, Korea.

[2]Department of Chemistry, Gangneung-Wonju National University, 7 Jukheon-gil, Gangneung-si, Gangwon-do, 25457, Korea.

*Correspondence to bjyoon@gwnu.ac.kr

I. Overall scheme of the HPLC system

Figure S1 shows a schematic diagram of the HPLC system that we developed for the experiments. The HPLC pump carried solvent (water) at a constant speed. Whenever a new experiment was performed after changing the temperature, the capillary loop was washed by injecting methanol several times. Upon injecting the sample, we stopped the HPLC pump and filled the sample into the injector loop. The pump speed was then increased gradually. The peristaltic pump pulls the sample (or washer) rather than pushes it to avoid contamination of the sample if the sample passed through the pump.

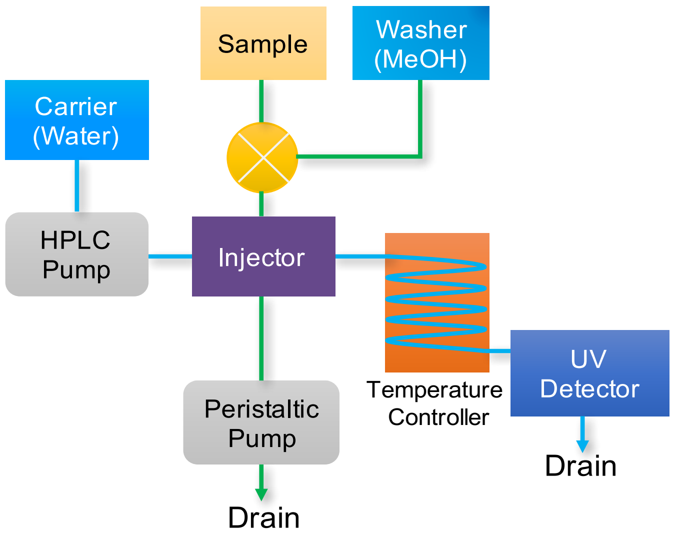

Figure S1. Schematic of the HPLC system. The capillary was actually rolled onto a round narrow copper block where the temperature was controlled. The yellow cross represents the three-way valve that changes the route to the injector from either the sample or the washer. All of the operations were automatically controlled by a computer program.

Other anomalies appearing from the peak-shape analyses with silica colloid sample are shown in Fig. S2. Figure S2a also shows the anomaly from the plot for the radius corresponding to the HPLC peak maximum found by using Eq. (10).

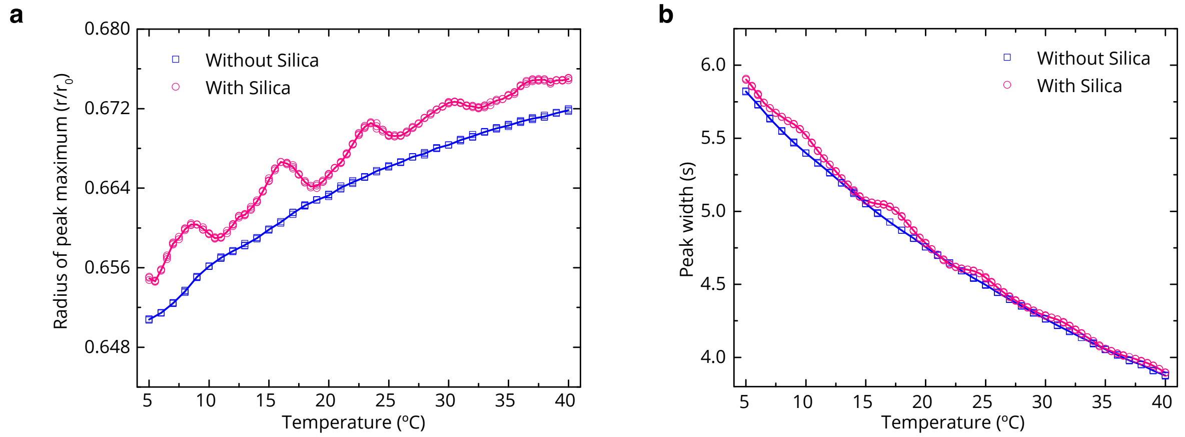

Figure S2. Other anomalies appearing from the peak-shape analyses. (**a**) The capillary radius corresponding to the HPLC peak maximum. The anomalous temperatures are the same as in Fig. 2a,b (text). (**b**) The peak full width at 90 % of the HPLC peak height. The pump speed was at 0.75 mL/min, and the silica colloid concentration was 1.5 wt %.

II. Analysis of the velocity profile

The velocity profile with respect to the radius in Fig. 1d was different from the typical laminar flow in a pipe that is described by a parabolic form. The velocity as a function of the radius is usually derived to the following Hagen-Poiseuille equation:

$$v(r) = -\frac{\Delta P/L}{4\mu} r^2 + A \ln r + C \,, \tag{s1}$$

where ΔP is the pressure difference between the two ends of the pipe and μ is the viscosity. The coefficient, A, is generally ignored by the boundary condition at the centre, and C is determined by the boundary condition of $v(r_0) = 0$ at the wall, assuming no slip. However, we found that the flow by an HPLC pump is greatly different from the flow driven by the pressure difference, ΔP, between two ends; i.e., the average velocity is determined by the pump speed and the high pressure is taken by the narrow UV cell or other compartments such as the back pressure regulator than the capillary loop. In other word, ΔP is considered to be zero in an HPLC flow. In Fig. S3, the velocity, first derivative, and second derivative with respect to the radius are plotted, and the value of the second derivative (Fig. S3c) was small except near the centre and wall. This implies that $\Delta P \cong 0$. The equations expressing the velocity and velocity derivatives for our experiment are shown in Eq. (s2-s4):

$$d^2v\,/dr^2 = A_1/(r+d)^2 + A_2/(1-r)^2, \tag{s2}$$

$$dv/dr = -A_1/\,(r+d) + A_2/\,(1-r) + B, \tag{s3}$$

$$v(r) = -A_1 \ln(r+d) - \;A_2 \ln\,(1-r) + \;Br + \;C. \tag{s4}$$

In these equations, the term d is introduced so the values do not diverge at the centre; the value of d is small but contributes importantly in Eq. (s3) and (s4). We define A_1 as a ‘draw force coefficient’ which is positive and A_2 as a ‘drag force coefficient’ which is negative. The draw force is produced by the fast flow at the centre driven by the HPLC pump and pulls the laminar layer by layer. The results agree well with the experimental data without an r^2 dependence in Eq. (s1). The coefficients, A_1, A_2, and B, fitted using Eq. (s3), and C fitted to Eq. (s4) are plotted in Fig. S4 along the temperature. Term B is not negligible and term C contributes substantially to the total velocity.

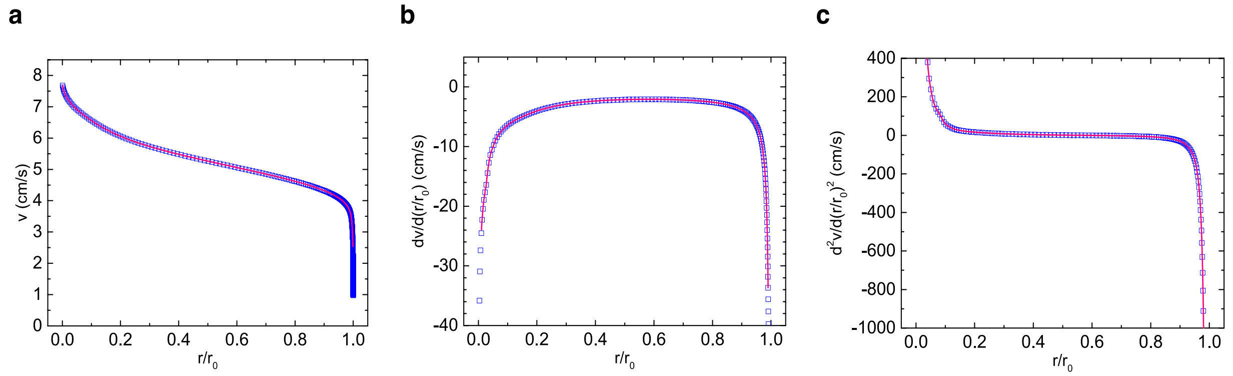

Figure S3. Velocity and its derivatives at 25 ˚C. (**a**) The velocity, (**b**) first derivative, and (**c**) second derivative. The blue symbols represent the experimental data, the red line in (a) is from Eq. (s4), (b) is from Eq. (s3), and (c) is from Eq. (s2). The pump speed was at 0.75 mL/min and the silica colloid concentration was 2.0 wt%.

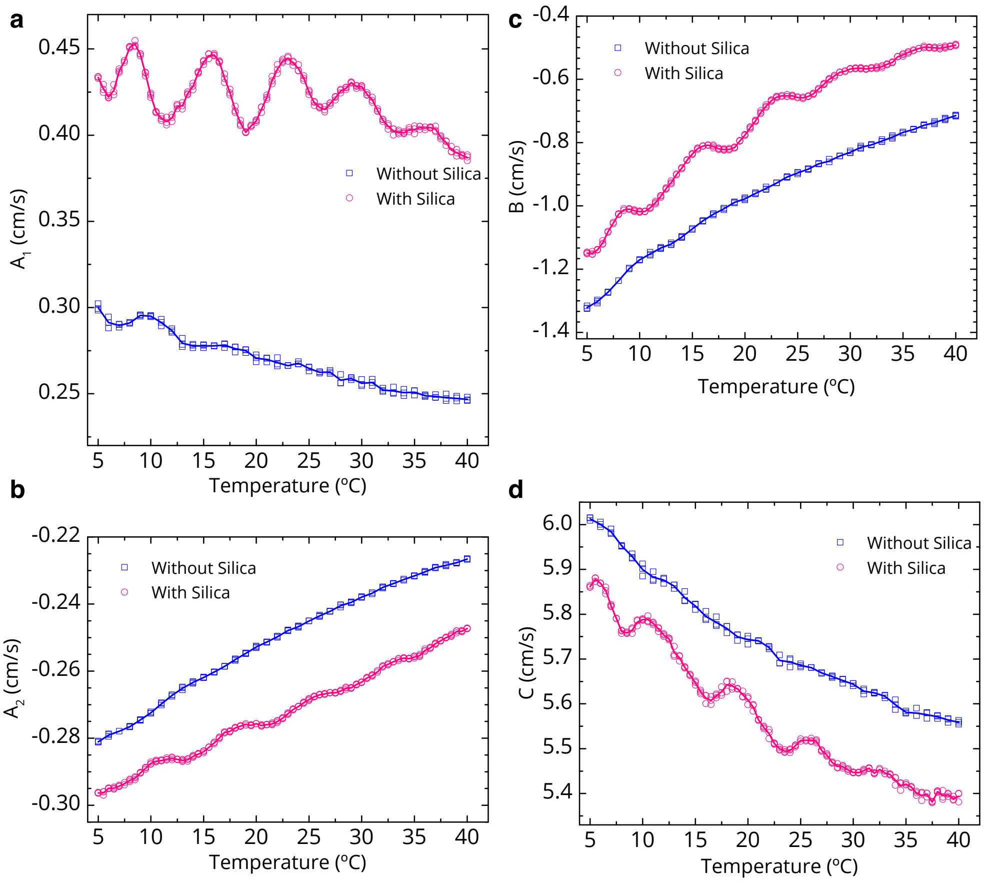

Figure S4. Coefficients A_1, A_2, B, and C. (**a-c**) The coefficients of A_1, A_2, and B were determined using Eq. (s3). (**d**) C was determined using Eq. (s4). The coefficients were obtained by fitting the experimental data at the 0.6 < r/r_0 < 0.9 region, where the anomaly appeared in the second derivatives, as in Fig. 1f. The AT was different at each radius, as like as it differed with the pump speed. However, the coefficients were unique for the selected region. Anomalies were present in the up-peaks in (a,c), and in the down-peaks in (b,d). The pump speed was at 0.75 mL/min.

The anomalous temperature (AT) behaviour appears in all of these coefficients when silica colloid is present in the sample. Among the coefficients, A_1 shows a great difference between the cases with silica and without silica. Specifically, the draw force coefficient, A_1, is related to the viscosity, and the values are high at the ATs. This indicates that the viscosity is high or the interaction between the layers is strong at the ATs. In Fig. S3, the fitted velocity, first derivative, and second derivative were plotted with red lines in good correspondence with experimental data. The exclusion zone (EZ) water[45] might be a kind of this anomalous specific vicinal water of high viscosity near solid interfaces rather than an *ad hoc* phase of water.

III. Experiments at low pump speeds

It is wonder in the text that the ATs were not observable at low pump speeds. In Fig. S5, the velocity gradients with respect to the radius ($-dv/d(r/r_0)$) are plotted with changing the pump speed. Those are linear substantially with pump speed. It was assumed that the thermal displacement increases incorporated with the velocity gradient. Thus, the extrapolation of the ATs to a zero pump speed with linear functions (Fig. 3) is considered to be valid according to Fig. S5.

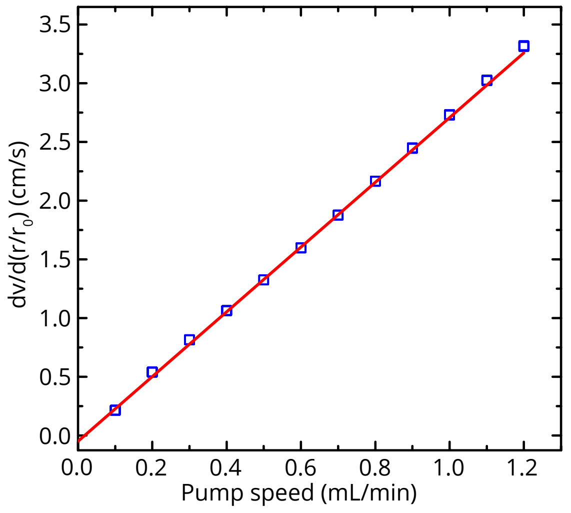

Figure S5. Velocity gradient at various pump speeds. The plot for the first velocity derivatives against pump speed at the radius corresponding to the HPLC peak maximum at 25 °C (without silica colloid) shows a good fit with a straight line. The negative sign was applied. Four data points are overlapped at the same pump speed.

The velocity difference between layers in the capillary flow involves essentially the need of energy and force to break the hydrogen bonds of water molecules. The energy is supplied by the HPLC pump. The force (F) involved by the velocity gradient is expressed with the following equation;

$$\frac{F}{A} = -\mu \frac{dv/r_0}{d(r/r_0)} . \quad \text{(s5)}$$

The force is calculated approximately as 7.8×10^{11} N/mol, when the following values are substituted; μ = 0.89 mPa·s at 25 °C; $dv/d(r/r_0) = -1.3$ cm/s (from Fig. S5 at a pump speed of 0.5 mL/min); r_0 = 0.025 cm; $A = 2\pi r_m \cdot \Delta l$ (r_m = 0.016 cm (from Fig. S2a), Δl = 2.8 Å/molecule). This value corresponds in magnitude to the force obtained roughly from the potential well of water[33,34], i.e., $\epsilon/\Delta l$ = 2400 R/2.8 Å ≅ 7.1×10^{11} N/mol, in which R is the gas constant. Therefore, it is expected that the force from the velocity gradient at the low pump speed is insufficient to excite the hydrogen bond, or to increase the displacement.

IV. Water–alcohol system

Water–alcohol mixtures are studied extensively and are also considered anomalous systems in their thermodynamics and structures[46,47]. The general behaviour of these systems is that the viscosity increases and the excess volume of mixing is negative in the mixtures. However, the partial molar volume of water in a water–ethanol mixture[48] or water–methanol mixture[49] increases slightly at water-rich concentrations. Although water–alcohol mixtures are understood to be homogeneous, the mixing is incomplete at the microscopic level[46]. The concentration of alcohol in this study was quite low, so the system was assumed to be less structured in the text. It is interesting that the ATs extrapolated to a zero pump speed were decreased by dissolving a small amount of alcohol; however, the observed ATs increased from those of pure water at high pump speeds because of the lower declining slope in Fig. 3c and d, as explained in the text. This presumably affects the body temperature differently in cells or in blood vessels during alcohol consumption.

Supplementary References

45. Pollack, G. H., *The fourth phase of water. Beyond solid, liquid, and vapor*. (Ebner & Sons Publishers, Seattle, 2013).
46. Franks, F. & Ives, D. J. G., The structural properties of alcohol-water mixtures. *Q. Rev. Chem. Soc.* **20**, 1−44 (1966).
47. Dixit, S., Crain, J., Poon, W. C. K., Finney, J. L. & Soper, A. K., Molecular segregation observed in a concentrated alcohol-water solution. *Nature* **416**, 829–832 (2002).
48. Manabe, M. & Koda, M., The partial molar volumes of normal chain alcohols in water-ethanol mixtures at 25 ˚C. *Bull. Chem. Soc. Jpn.* **48**, 2367−2371 (1975).
49. Soetens, J. -C. & Bopp, P. A., Water-methanol mixtures: Simulations of mixing properties over the entire range of mole fractions. *J. Phys. Chem. B* **119**, 8593−8599 (2015).
